# Supplementary material for: Epigenetic Priming with Decitabine Augments the Therapeutic Effect of Cisplatin on Triple-Negative Breast Cancer Cells through Induction of Proapoptotic Factor NOXA
Source: Cancers (Basel). 2022 Jan 4;14(1):248. doi: 10.3390/cancers14010248 (PMC8749981; doi:10.3390/cancers14010248)
Supplement: Supplementary file 1 [file cancers-14-00248-s001.zip › cancers-1489272-supplementary.pdf]

Supplementary Information for

Epigenetic priming with decitabine augments the therapeutic effect of cisplatin on triple-negative breast cancer cells through induction of proapoptotic factor NOXA

Nakajima et al.

Supplementary Figure 1

(Figure1e)

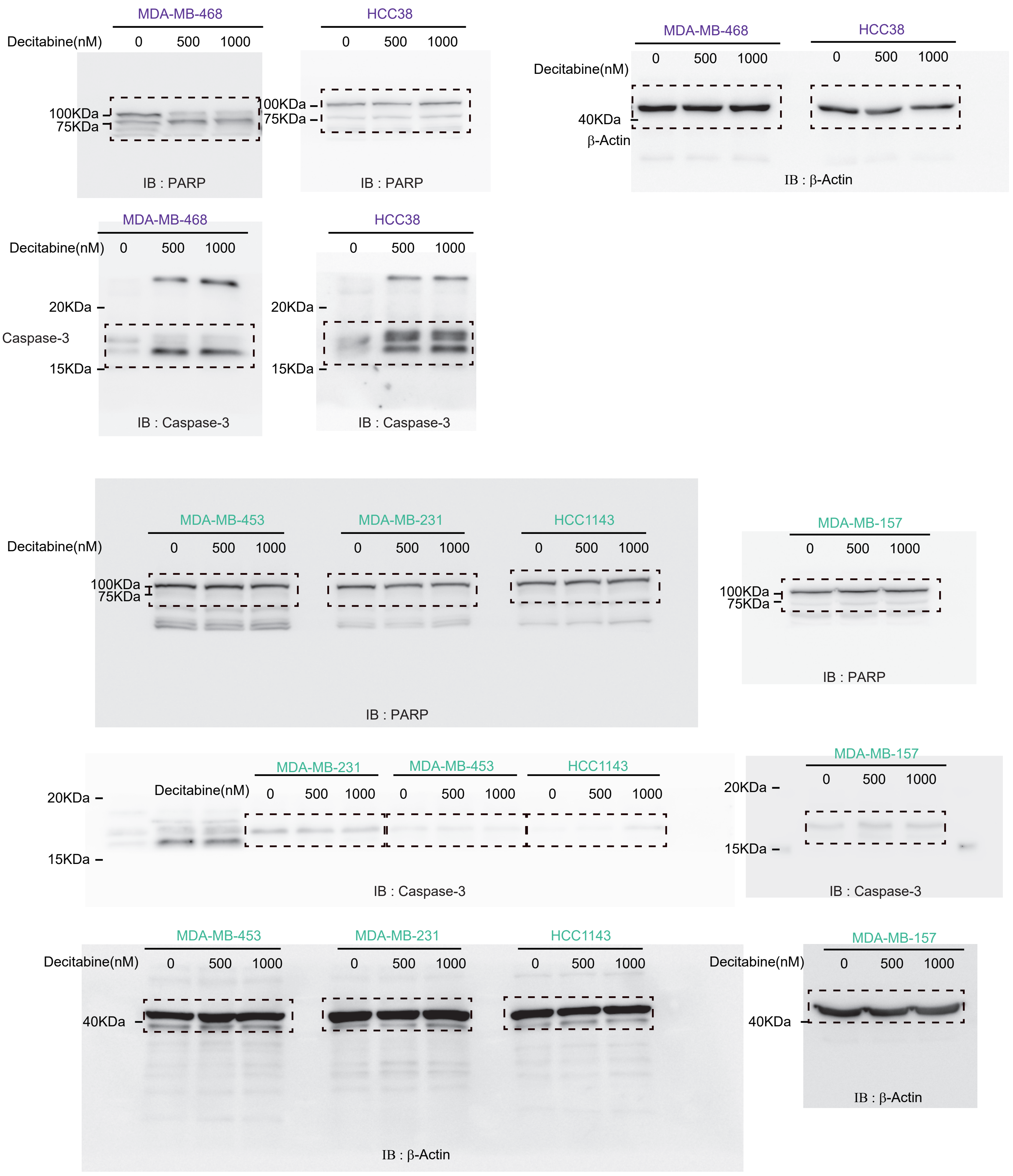

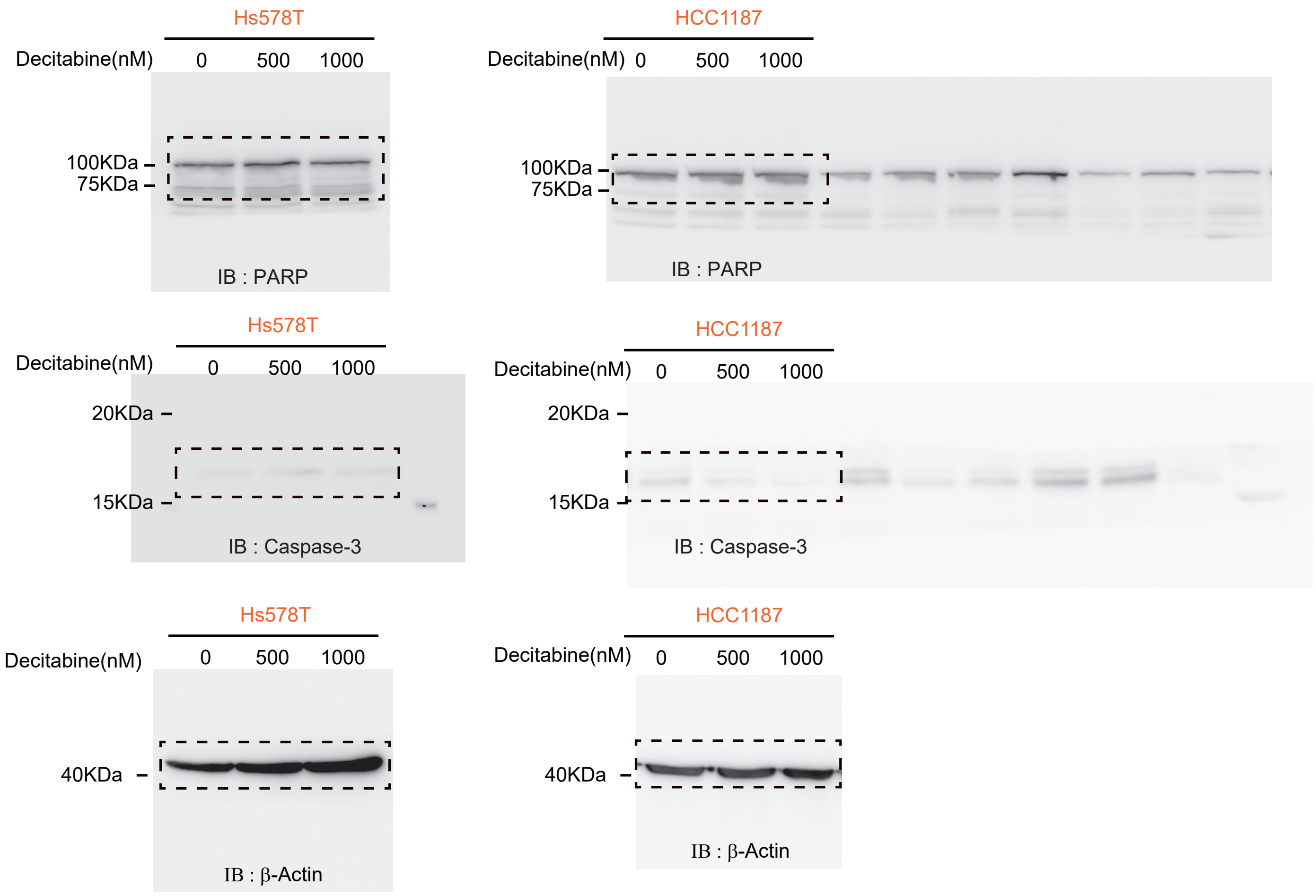

Figure S1. Raw data of Immunoblot from Fig 1.

Supplementary Figure 2

(Figure3d)

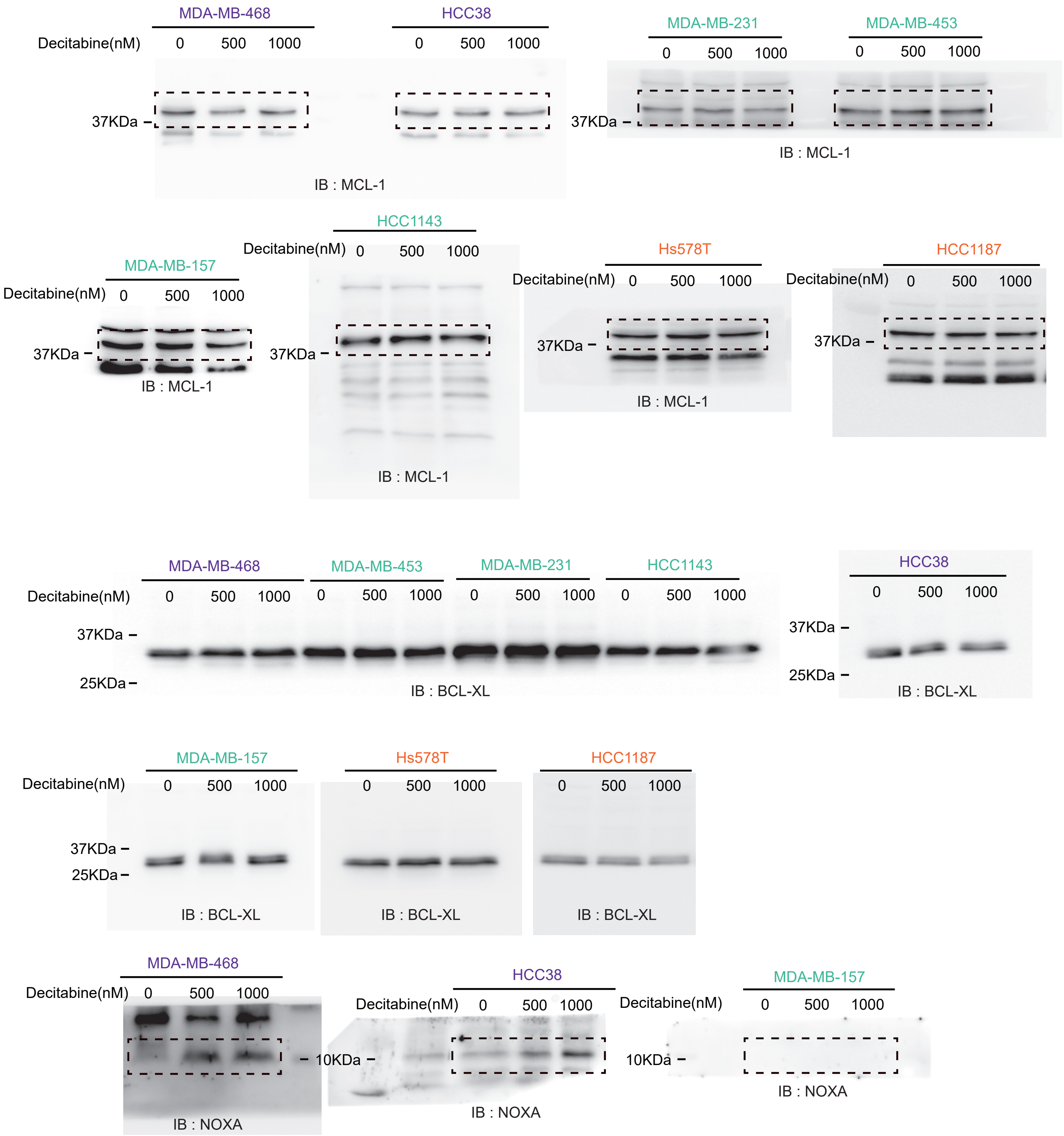

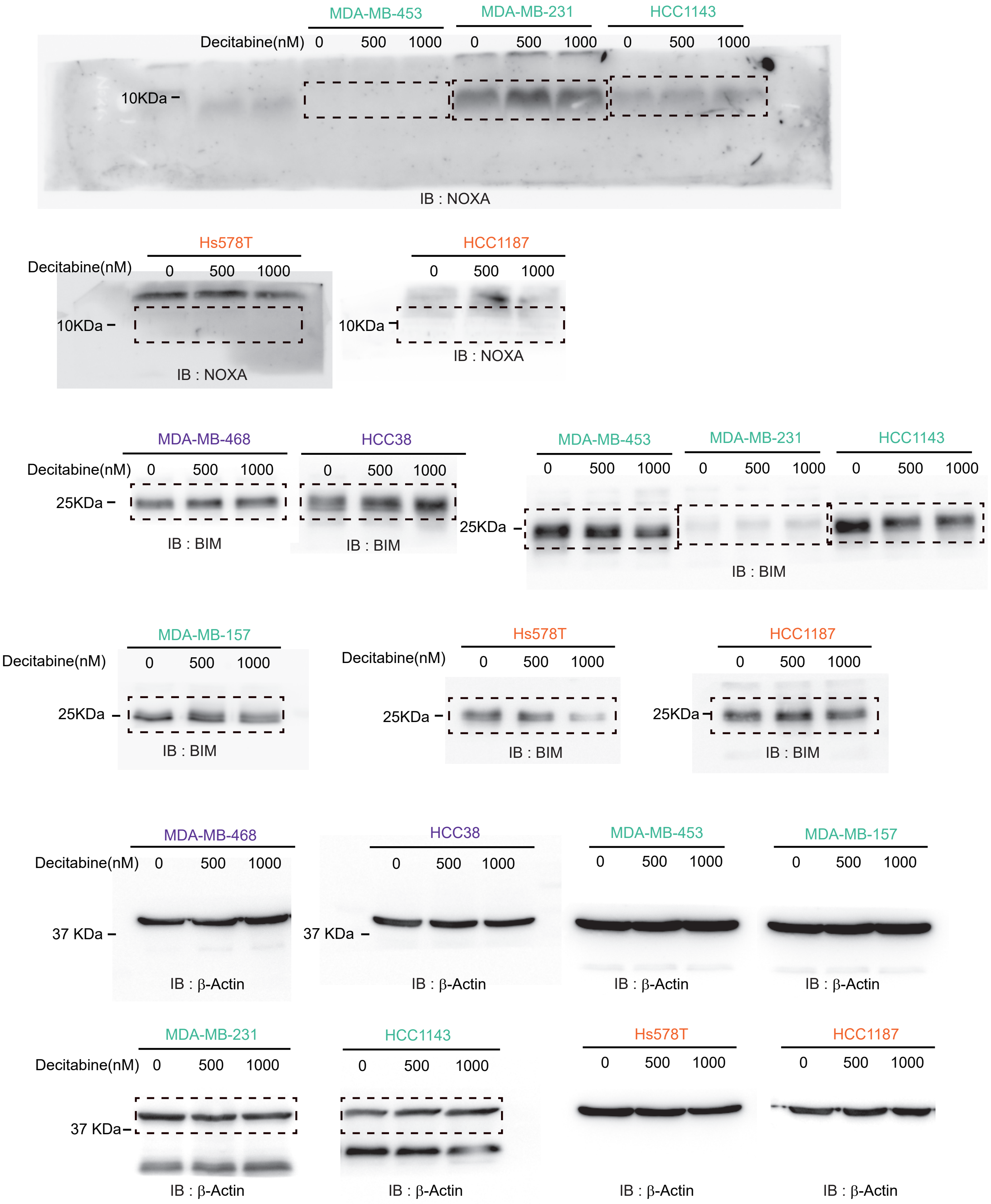

Figure S2. Raw data of Immunoblot from Fig 3.

Supplementary Figure 3

(Figure4c)

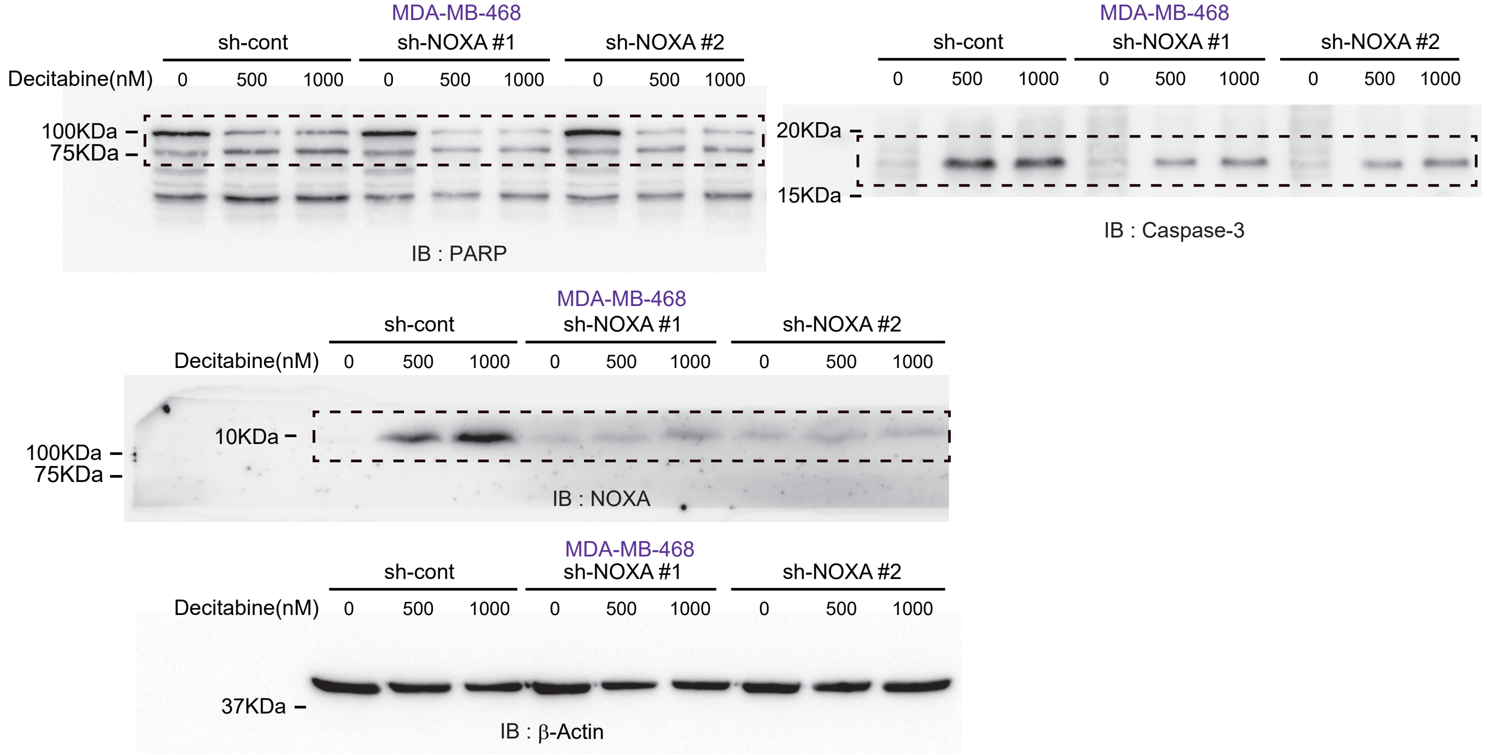

(Figure4f)

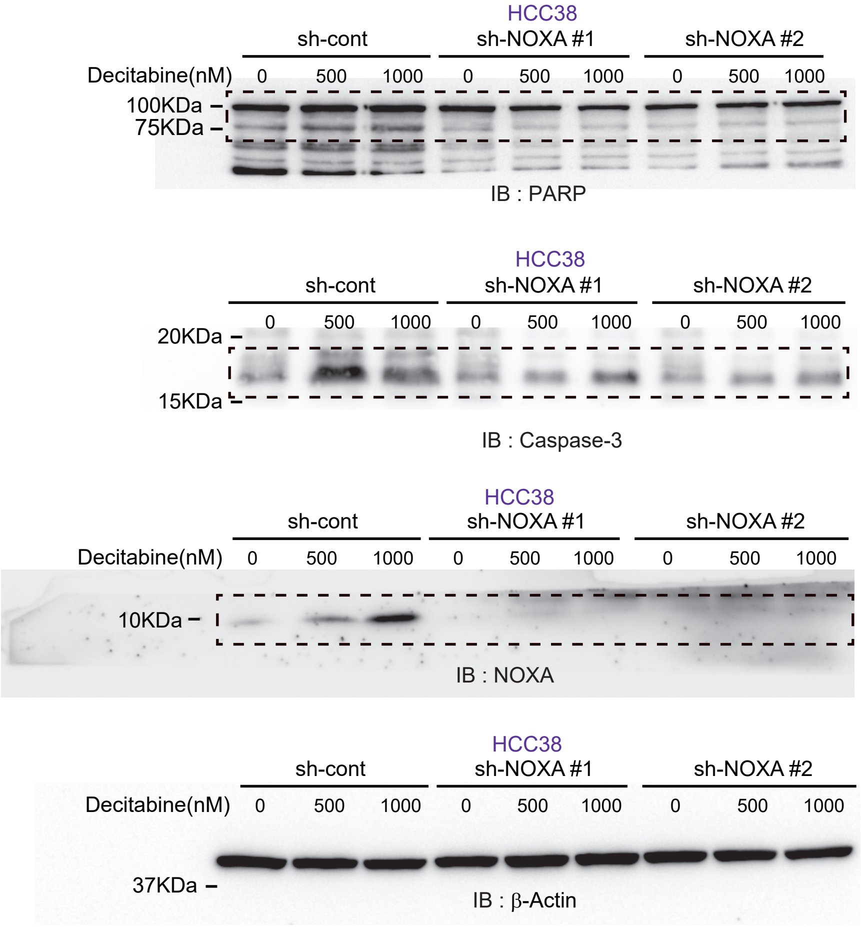

Figure S3. Raw data of Immunoblot from Fig 4.

Supplementary Figure 4

(Figure5b)

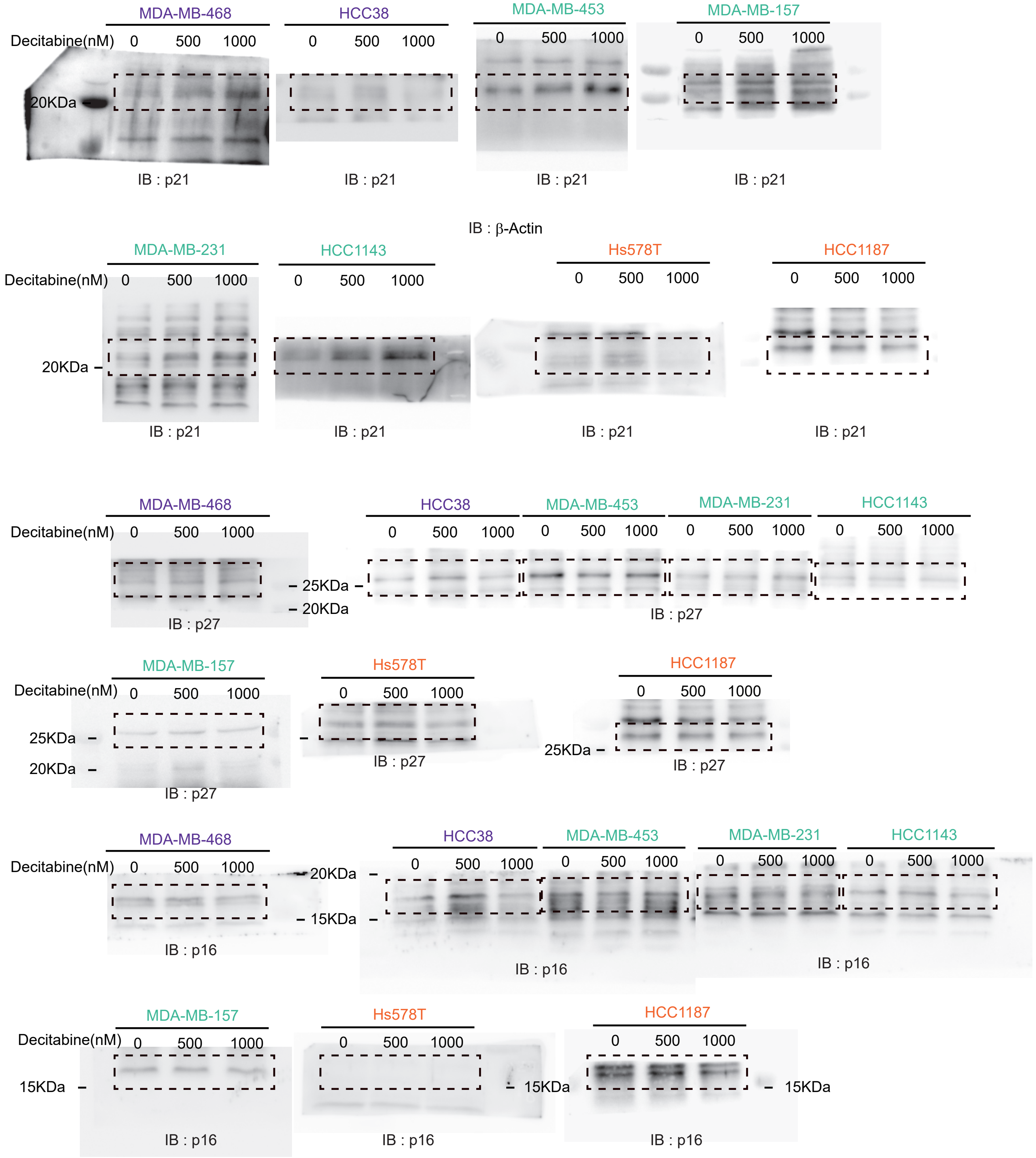

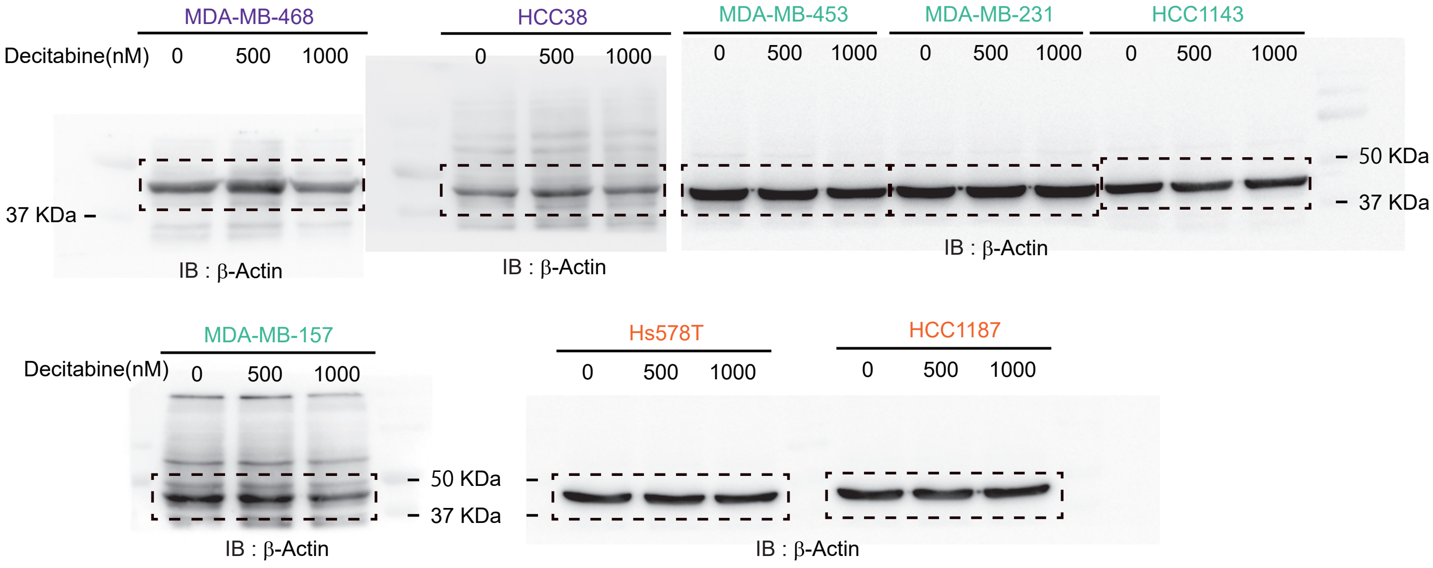

Figure S4. Raw data of Immunoblot from Fig 5.

Supplementary Figure 5

(Figure6a)

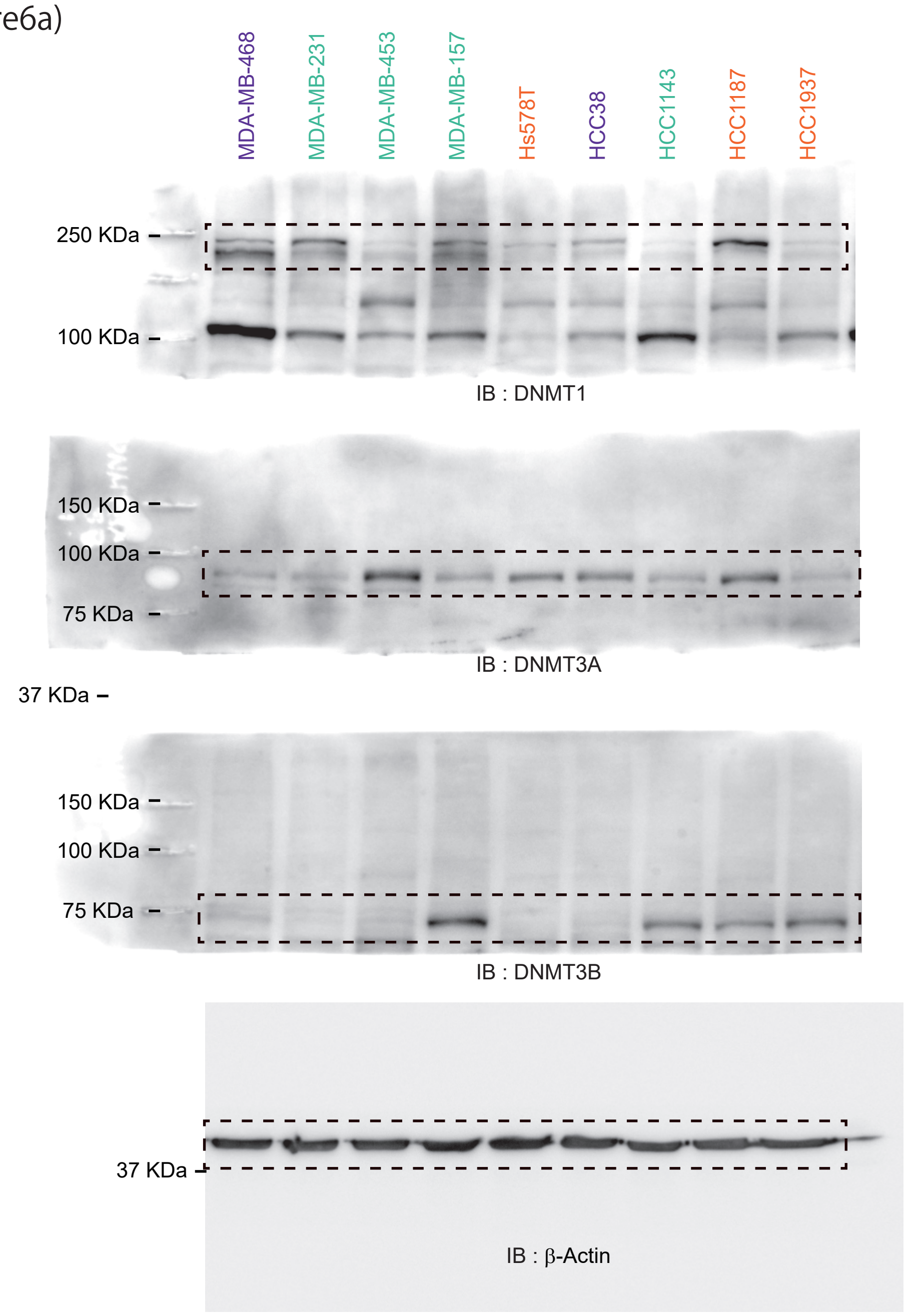

(Figure6b)

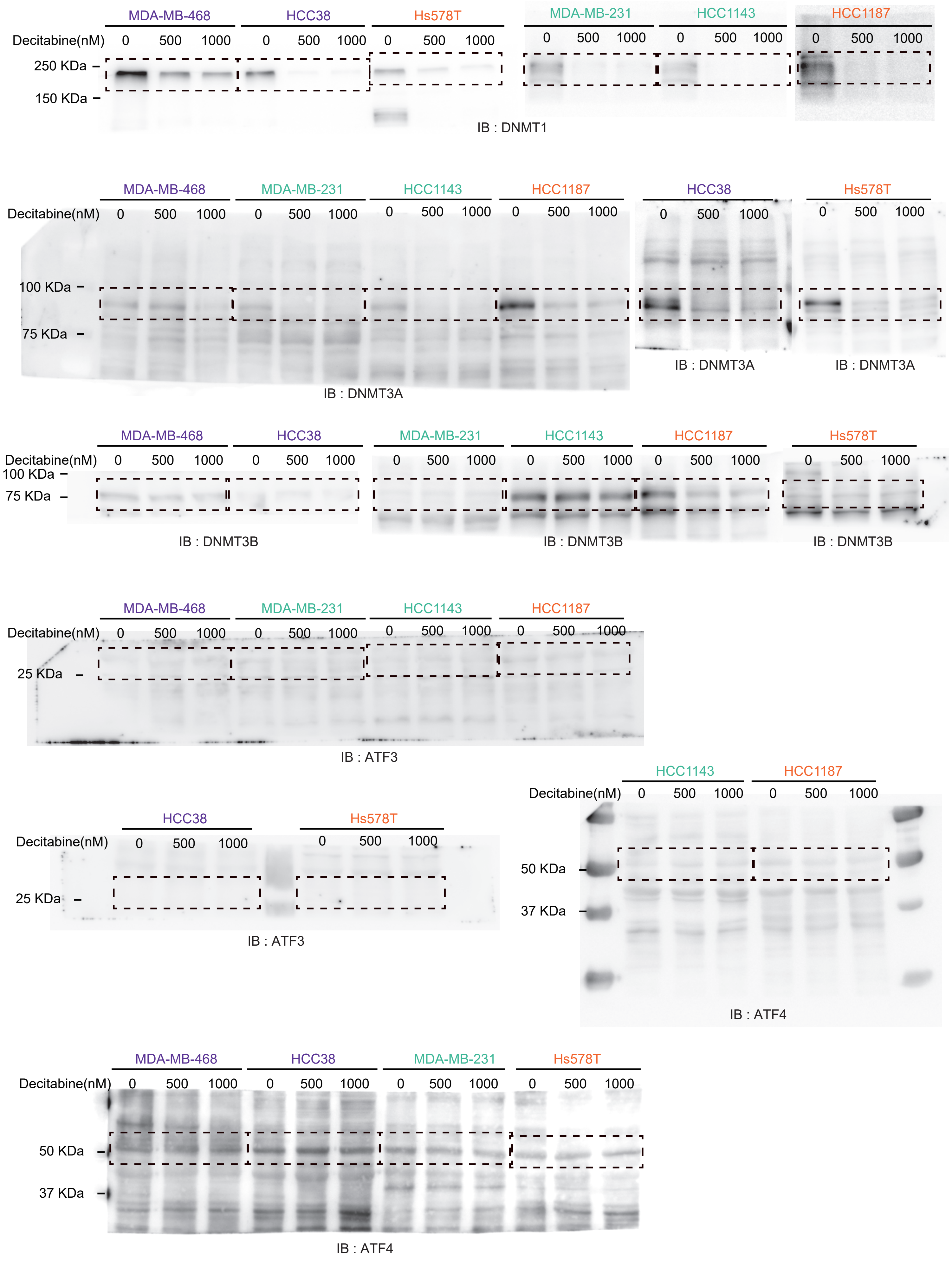

MDA-MB-468
HCC38
Hs578T
MDA-MB-231
HCC1143
HCC1187
Decitabine(nM) 0 500 1000 0 500 1000 0 500 1000 0 500 1000 0 500 1000 0 500 1000
250 KDa
150 KDa
IB : DNMT1
MDA-MB-468
MDA-MB-231
HCC1143
HCC1187
HCC38
Hs578T
Decitabine(nM) 0 500 1000 0 500 1000 0 500 1000 0 500 1000 0 500 1000 0 500 1000
100 KDa
75 KDa
IB : DNMT3A
IB : DNMT3A
IB : DNMT3A
MDA-MB-468
HCC38
MDA-MB-231
HCC1143
HCC1187
Hs578T
Decitabine(nM) 0 500 1000 0 500 1000 0 500 1000 0 500 1000 0 500 1000 0 500 1000
100 KDa
75 KDa
IB : DNMT3B
IB : DNMT3B
IB : DNMT3B
MDA-MB-468
MDA-MB-231
HCC1143
HCC1187
Decitabine(nM) 0 500 1000 0 500 1000 0 500 1000 0 500 1000
25 KDa
IB : ATF3
HCC38
Hs578T
Decitabine(nM) 0 500 1000 0 500 1000
25 KDa
IB : ATF3
HCC1143
HCC1187
Decitabine(nM) 0 500 1000 0 500 1000
50 KDa
37 KDa
IB : ATF4
MDA-MB-468
HCC38
MDA-MB-231
Hs578T
Decitabine(nM) 0 500 1000 0 500 1000 0 500 1000 0 500 1000
50 KDa
37 KDa
IB : ATF4

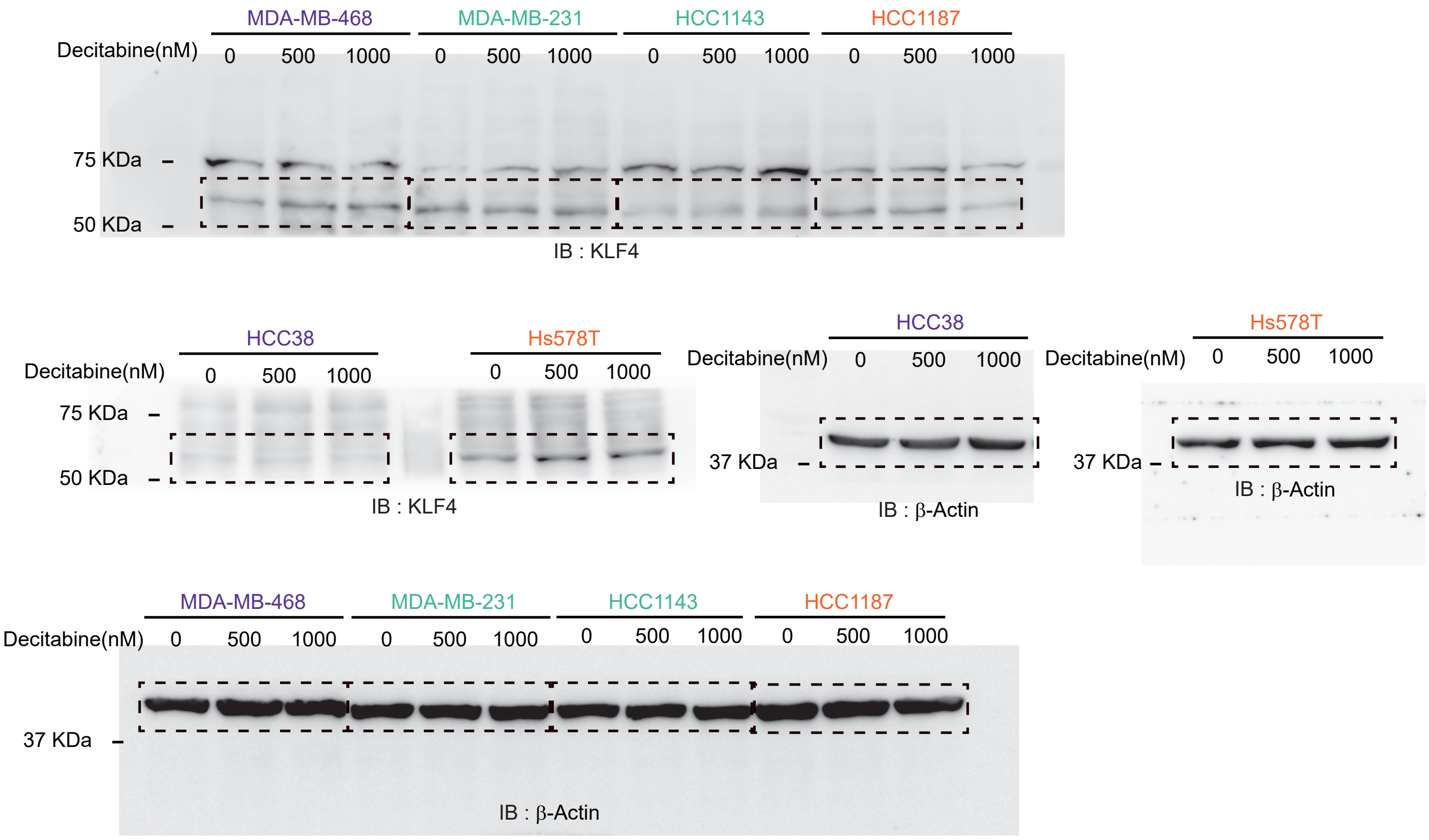

Figure S5. Raw data of Immunoblot from Fig 6.

Supplementary Figure 6

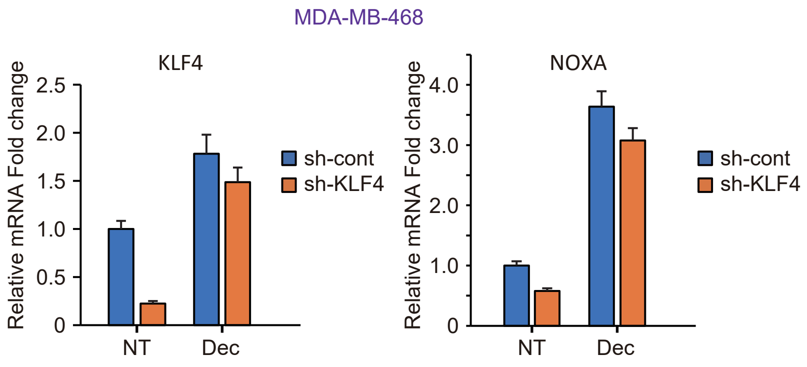

Figure S6. KLF4 did no effect on the induction of NOXA by decitabine. MDA-MB-468 cells were infected with shRNAs (sh-control and sh-KLF4). Cells were exposed to 500 nM decitabine. After treatment for 72 h, mRNA levels were measured by quantitative real-time PCR (qPCR).
Error bars indicate SD (n=3).

Supplementary Figure 7

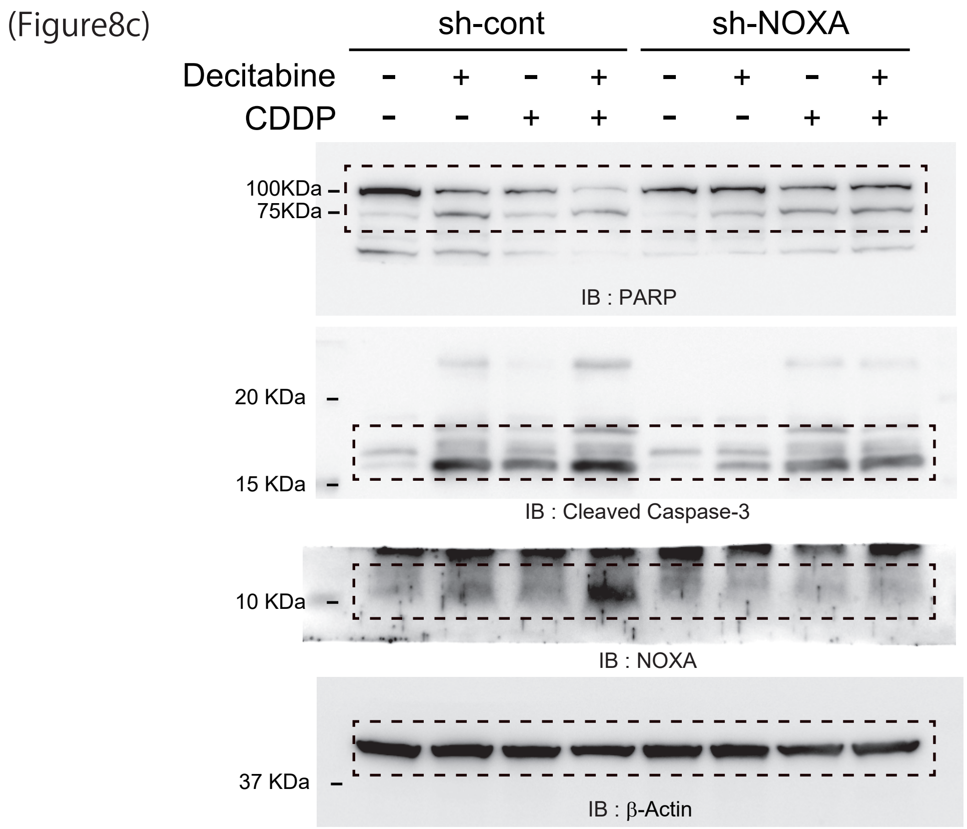

Figure S7. Raw data of Immunoblot from Fig 8.
